# Development of a One-Step Multiplex Real-Time PCR Assay for the Detection of Viral Pathogens Associated With the Bovine Respiratory Disease Complex

**DOI:** 10.3389/fvets.2022.825257

**Published:** 2022-01-26

**Authors:** Juan Zhang, Wan Wang, Mujiao Yang, Jun Lin, Fei Xue, Yuanmao Zhu, Xin Yin

**Affiliations:** State Key Laboratory of Veterinary Biotechnology, Division of Livestock Infectious Diseases, Harbin Veterinary Research Institute of Chinese Academy of Agricultural Sciences, Harbin, China

**Keywords:** BRDC, IDV, BVDV, BoHV-1, BRSV, BPIV3, quantitative PCR

## Abstract

Bovine respiratory disease complex (BRDC) occurs widely in cattle farms. The main viral pathogens include bovine viral diarrhea virus (BVDV), Bovine herpesvirus 1 (BoHV-1), bovine parainfluenza virus type 3 (BPIV3), and bovine respiratory syncytial virus (BRSV), and the newly emerged influenza D virus (IDV). In this study, we have developed a one-step multiplex real-time Polymerase Chain Reaction (PCR) capable of simultaneously detecting these five viral pathogens causing BRDC. The established assay could specifically detect targeted viruses without cross-reaction with others. The detection limit was ~10 copies/reaction for single real-time PCR and 100 copies/ reaction for multiplex real-time PCR assay. A total of 213 nasal samples from cattle with signs of respiratory tract disease were then collected for performance evaluation of the established platform, proving that the method has good specificity and sensitivity. The surveillance data suggested that BVDV and BoHV-1 infections are the dominant cause of BRDC in the herd, whereas the detection rate of IDV, BIPV3, and BRSV is relatively lower. In summary, the established assay provides technical support for rapid clinical detection of BRDC associated viral pathogens to guide the formulation of BRDC prevention and control measures.

## Introduction

Bovine respiratory disease complex (BRDC) has posed a great threat to the dairy and beef industries throughout the world (1). The disease is usually resulted from stress, primary viral infection and secondary bacterial infection, leading to high mortality and morbidity in cattle (2, 3). It's considered that single or combinational infections by viral pathogens are the prerequisites to developing BRDC (4). So far, the viral pathogens including Bovine Viral Diarrhea Virus (BVDV), Bovine herpesvirus 1 (BoHV-1), Bovine Parainfluenza Virus Type 3 (BPIV3), Bovine Respiratory Syncytial Virus (BRSV) and Influenza D Virus (IDV) have been proposed to be directly associated with BRDC (5). Moreover, secondary bacterial infections such as *Mannheimia haemolytica, Pasteurella multocida*, and *Haemophilus somnus* during or after viral infections potentially cause fatal complication of viral infections (6, 7). Thus, early diagnosis of viral pathogens associated with each outbreak could be beneficial to timely managing and controlling BRDC (1).

BVDV is single-stranded, positive-sense RNA viruses that belongs to genus *Pestivirus*, family *Flaviviridae* (8). BVDV are genetically divided into genotype 1 (BVDV-1), genotype 2 (BVDV-2) and genotype 3 (BVDV-3) (9). Previous studies have concluded that BVDV aggravates the respiratory disease syndrome by causing immune tolerance and persistent infection (10). BoHV-1 harbors a linear double-stranded DNA genome and causes infectious bovine rhinotracheitis (IBR), a highly contagious infectious respiratory disease (11, 12). The World Organization for Animal Health (OIE) lists IBR as a Category B animal disease. As reported, BoHV-1 preferentially infects the basal epithelium of injured monolayers and/or genital tracts to induce mucosal barrier injury (13, 14). Serological survey of BoHV-1 infection in China suggested that the seropositive rate was approximately 40% (11). Unlike BoHV-1, BPIV3 is restricted to replicate in the apical ciliated epithelium causing tissue damage and immunosuppression which often progresses to bronchial pneumonia in calves and adult cattle (15). BRSV infection is the major cause of BRDC in calves during their first year (16). It infects both ciliated bronchial epithelia and type II pneumocytes, and causes minimal to extreme respiratory diseases (17). In addition to these well-known viral pathogens, IDV, a new type of influenza virus with cattle as a primary reservoir, can infect and cause influenza diseases in cattle (18). Recently, IDV was reported as a crucial viral agent that drives the occurrences of bovine respiratory disease syndrome (18). However, the contribution of IDV to the bovine respiratory disease development as well as the co-infection between IDV and other respiratory viral pathogens remains unknow. Due to the similarity of clinical signs and co-infections involved in BRDC, it's nearly impossible to rapidly and accurately identify the causative agents solely based on the clinical signs and conventional methods such as pathogens isolation. Therefore, single and multiplex real-time PCR assays have been developed for the rapid detection of pathogens associated with BRDC in the past years (13, 17, 19–21). However, the single target real-time PCR requires separate amplification by consuming excess resources along with inefficient processing. Direct diagnostic methods assay for the presence of the viruses associated with BRDC are still lacking. In addition, the relative contribution of these viruses in BRDC development has not been defined yet, especially in China (22). In this study, we developed a multiplex real-time PCR assay for synchronously detecting five viral pathogens associated BRDC. We showed that the optimized assay displayed excellent performance. The detection limits for all these five viral pathogens were 100 copies/reaction with the multiplex real-time PCR assay. More importantly, our established assay can effectively detect BRDC related pathogens from the nasal swabs collected from the cattle with signs of respiratory tract disease.

## Materials and Methods

### Viruses and Cell Cultures

BVDV-1a NADL strain (ATCC VR-534), Bovine rotavirus (BRV) strain NCDV (ATCC VR-452) *Pasteurella multocida* P-1059 (capsular serogroup A) were obtained from China Veterinary Culture Collection Center (CVCC). BoHV-1 isolate HLJ07, BRSV field isolate HLJ01, Bovine coronavirus (BCoV) isolate HM and *Mannheimia haemolytica* SH1801 (Serotype 1), and BPIV-3 (HQ530153) were isolated by our laboratory (23). BVDV-2 HLJ-10 strain was provided by Dr. Mingchun Gao at Northeast Agricultural University (24). The Nucleic Acid of CSFV vaccine strain (C-strain) was provided by Dr. HuaJi Qiu at Harbin Veterinary Research Institute of Chinese Academy of Agricultural Sciences (25). BVDV, BoHV-1, BPIV3 were propagated on Madin-Darby bovine kidney (MDBK) cells. BRSV was propagated on bovine Turbinate (BT) cells cultured in Minimum Essential Medium (MEM) (Thermo Fisher, Inc.; USA) supplemented with 2% bovine serum (GE, Inc.; USA). IDV strain D/bovine/Mississippi/C00046N/2014 was obtained via reverse genetics system and propagated on Madin-Darby Canine Kidney (MDCK) cells (26).

### Primer and Probe Design

All available sequences of BVDV, BoHV-1, BRSV, BPIV3 and IDV were retrieved from GenBank for alignment via MEGA X. To obtain the specific primer/probe set for the assay development, we selected the highly conserved regions BVDV 5'UTR region, BoHV-1 glycoprotein E (*gE*) gene, BRSV nucleocapsid (*N*) gene, BPIV3 matrix (*M*) gene, IDV *PB1* gene for primer/probe design using the Oligo7 and Primer Express 3.0.1 software. The designed primer/probe sets were then subjected to Primer-BLAST for specificity validation. The primer/probe sets with excellent specific properties were finally chosen for the assay development. All oligonucleotides listed in Table 1 were synthesized by RuiBiotech (Beijing, China).

**Table 1:** Primer and probes used in this study.

**Pathogens**	**Primer or probes**	**Sequence (5'-3')**	**Position**	**Length**
BVDV	BVDV-F228	TCGAGATGCCACGTGGAC	228–245	162 bp
	BVDV-UTR	ATGTGCCATGTACAGCAGA	371–390	
	BVDV-Pro	CY5-ACCCTATCAGGCTGT-MGB	322–337	
BOHV-1BoHV-1	gE-85bp-F	CCGCCAATAACAGCGTAGA	122735–122753	85 bp
	gE-85bp-R	CCGTTGTACTGCAGCACAA	122801–122819	
	BOHV-1-gE-Pro	FAM-CCTCCGGGCTTTAC-MGB	122783–122796	
BRSV	BRSV-N-141bp-F	ATACAAAGGACTCATCCCGAAAG	155–173	75 bp
	BRSV-N-141bp-R	AAGATTCCTTCTACCCTACTACCTCC	201–220	
	BRSV-N-Probe	NED-AGTATTTGAAAAGTACCCTC-MGB	178–192	
BPIV3	BPIV3M-113bp-F	CAGGAACTCCTACAAGCCGC	164–183	113 bp
	BPIV3M-113bp-R	CATGGGTACAGTTCAGGTTTAATG	317–338	
	BPIV3-MTPro	VIC-CTATCATCTCCGTGGC-MGB	218–237	
IDV	IDV-F (19)	AATTCTGTGCCAATGAAGCTG	320–340	104 bp
	IDV-R (19)	TGGCATATTTCTTTCACTTGTCC	401–423	
	IDV-Pr	ROX-CATAAGTTTGYCTTCCTTCAGTG-MGB	375–397	

### Nucleic Acid Extraction

DNA/RNA was extracted from cell cultures or clinical samples following the instruction of Axygen Body Fluid Viral DNA/RNA Miniprep Kit. Briefly, 2 mL of DMEM was added into the nasal swabs and mixed well. After centrifugation at 5,000 rpm for 5 min, the clarified supernatants were then collected and used for nucleic acid extraction. To obtain the DNA/RNA from either clinical samples or infected culture fluids, 200 μL of the supernatants were mixed with equal amount of lysis buffer. After incubation at room temperature for 5 min, the mixture was mixed with 75 μL of V-N buffer. The supernatants were then collected after centrifugation for RNA extraction. Finally, the resulting RNA was eluted using 30 μL elution buffer and stored at −80 °C for further use.

### Virus Titration on Cell Cultures

BVDV, BoHV-1, and BPIV3 were propagated in MDBK cells. Briefly, the monolayer MDBK cells were inoculated with the indicated virus stock at MOI of 0.02. After one hour adsorption, the cultures were rinsed and washed three times with PBS. DMEM supplemented with 2% horse serum was then added for maintenance. The cell cultures are collected once 100% cytopathic effect (CPE) is observed. The TCID_50_ values were determined using standard methods after incubation for 48–120 h.

### Sensitivity Test

To determine the lowest detection limit of primers and probes used in this study, the specificity tests of single real-time PCR and multiple real-time PCR were conducted in single tube. Liner positive control with the copy number ranging from 10^7^ to 10^1^ was used as standards.

### Real-Time PCR

The fixed reaction conditions for the multiplex reaction system were as follow: 10 μL of 2 × one step RT-PCR buffer III (Takara), 0.4 μL of Ex Taq HS, 0.4 μL of PrimeScript RT Enzyme Mix II, 0.1 μL of each primer set (10 μmol), 0.2 μL of Probe (10 μmol), 2 μL of nucleic acid template, and enzyme-free water 5.7 μL. Amplification was carried out using the following program: 42°C, 5 min, 95°C, 10 s, 45 cycles of 95°C 5 s, 60°C 30 s.

### Clinical Samples

A total of 213 nasal swab samples were collected from the cattle with signs of respiratory tract disease in the northeastern part of China. Among them, 30 nasal swabs were collected from dairy herds in 2019, 38 nasal swabs were collected from dairy herds in 2020, while the remaining 145 nasal swabs were collected from dairy herds in 2021. Most these sampled animals had either one or several of the following symptoms: fever >39 °C, cough, serious nasal and/or lacrimal discharge, and breath sounds. The nasal samples were stored frozen at −80°C until use.

## Results

### Analytical Specificity of the Primer/Probe Sets Used in the One-Step Real-Time RT-PCR Assay

**Figure 1:**
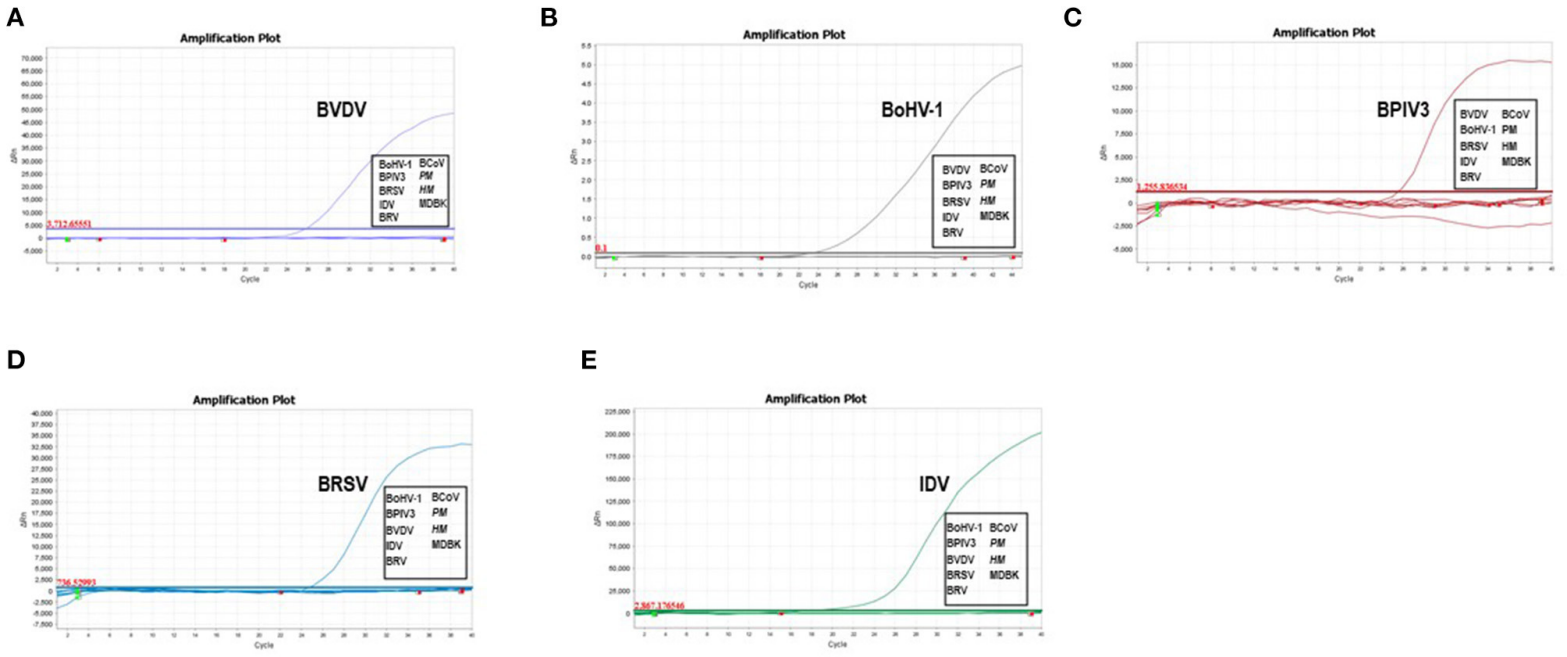
Analytical specificity of the primer/probe sets used in the one-step real-time RT-PCR assay: Amplification curves represent samples positive for BVDV, BoHV-1, BPIV3, BRSV and IDV detected by the real-time qRT-PCR assay. Negative samples include BCoV, BRV, *Pasteurella multocida* and *Mannheimia haemolytica*. Equal amount of isolated viral/bacterial genome were mixed for the assay. The non-detected samples were highlighted in the box.

To experimentally evaluate the specificity of the selected primer/probe sets, we isolated the viral genomes from the cultured viruses including BVDV NADL strain, BoHV-1 isolate HLJ07, BPIV3 strain HQ510351, BRSV field isolate HLJ01, IDV strain D/bovine/Mississippi/C00046N/2014, BCoV isolate HM and BRV strain NCDV as well as the bacterial genomes from *Pasteurella multocida* and *Mannheimia haemolytica* for testing. We found that the designed primer/probe set could successfully recognize and amplify the gene fragment derived from the corresponding virus. No amplification and fluorescent signals were observed for non-related pathogens (Figures 1A–E). These results proved that each set of primers and probes exhibited good specificity which can be applied for the assay development.

### Establishment of the Standard Curve for the Multiplex Real-Time PCR

**Figure 2:**
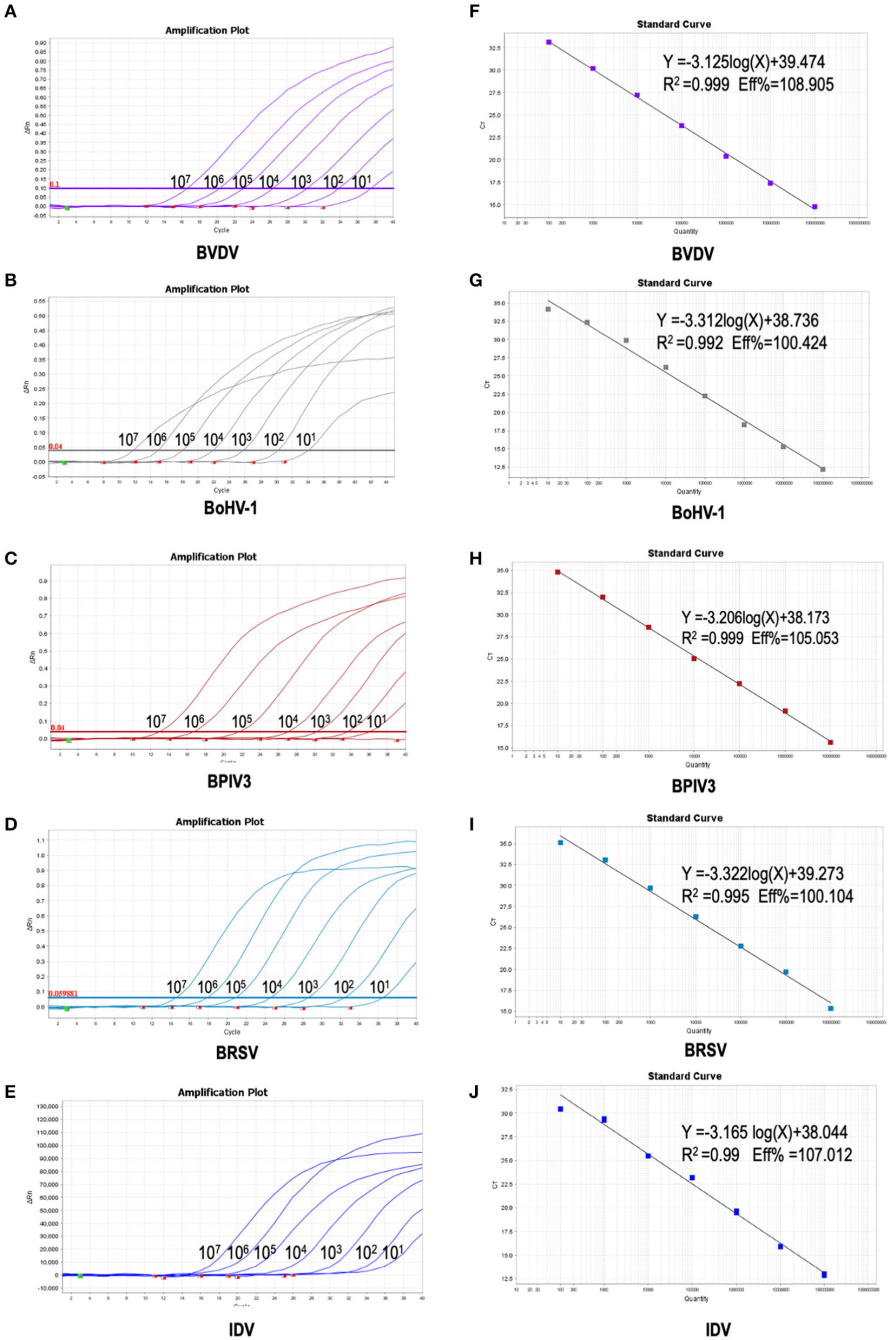
Preparation of plasmid standards: **(A–E)**: amplification curves (X-axis: Cycle, Y-axis: ΔRn) of BVDV, BPIV3, BRSV, BoHV-1 and IDV for each plasmid standard with concentrations ranging from 1 ×10^7^copies/μL to 1 × 10^1^copies/μL; **(F–H)**: standard curves of plasmid standards of BVDV, BPIV3, BRSV, BoHV-1and IDV. **(F)**: BVDV: Y = −3.125log(X)+39.474 R^2^ = 0.999 Eff% = 108.905; **(G)**: BoHV-1: Y = −3.312log(X)+38.736 R^2^ = 0.992 Eff% = 100.424; **(H)**: BPIV3: Y = −3.206log(X)+38.173 R^2^ = 0.999 Eff% = 105.053; I: BRSV: Y = −3.322log(X)+39.273 R^2^ = 0.995 Eff% = 100. 104; **(J)**: IDV: Y = −3.165 log(X)+38.044 R^2^ = 0.99 Eff% =107.012.

To further verify the sensitivity of the multiplex detection method, we generated the recombinant plasmids containing the five target fragments amplified from IBPV3 [*M* gene (4039-4194)], BoHV-1 [*gE* gene (120290-123438)], BVDV-1b [*5'-UTR* (64-494)], BRSV [*N* gene (1342-2289)], and IDV [*PB1*gene (129-488)] and cloned into pLVX-IRES-zsGreenI vector created by Clontech Laboratories Inc. The primers used for amplification were shown in Table 2, respectively. The standard curves for the five detected viruses were then determined using a range of 10-fold diluted recombinant plasmids with concentrations from 10^7^ to 10^1^ copies/μL. The qPCR amplifications of each standard plasmid DNA showed amplification plots corresponding to mean Ct values of 16.272–35.966 for BVDV, 15.697–35.694 for BoHV-1, 17.563–36.402 for IBPV3, 20.394–39.084 for BRSV, 17.385–36.634 for IDV (Figures 2A–E left panel). The slope of the standard curve, correlation coefficient (R^2^), and amplification efficiency (Eff%) were calculated as follows: −3.125, 0.999, and 108.905 % for BVDV; −3.312, 0.992, and 100.424% for BoHV-1, −3.206, 0.999, and 105.053% for BPIV3, −3.322, 0.995, and 100.104% for BRSV, −3.165, 0.990, and 107.012% for IDV (Figures 2A–E right panel), showing an excellent amplification efficiency and linear equation required for RNA quantification.

**Table 2:** Primers used for standard constructions.

**Pathogens**	**Primer or probes**	**Sequence (5'-3')**	**Position**	**Length**
BVDV	BVDV-F54	CCGGAATTCCGGGACAAATCCTCCTTAGCGAA(EcoRI)	64–85	450 bp
	BVDV-2F54	CGC ctcgag TCTCCTCTCTCGCCAAACA(XhoI)	476–494	
BOHV-1	BOHV-1-gI-gE-L	gttaTCTAGAgtgatggtgatggtgatgGCGGAGGATGGACTTGAGTCG(xba I)	120290–120309	3188 bp
	BOHV-1-g3E-U	GATC gactagt CGGTGCCTGTTGCTCTGGAT(Spel I)	123418–123438	
BRSV	BRSV-NF2	GATCGACTAGTC GCCACCATG ATGTTATATGCTATGTCCCGAT (Spel I)	200–221(N)	980 bp
	BRSV-NR2	GTTA CTCGAG CCAATTGGTTCTTGATTGCCTC (Xhol)	1127–1147(N)	
BPIV3	BPIV3 MF1	CTAG TCTAGAAACGAACAAAGGAAGGCAAT(XbaI)	3834-3853	881 bp
	BPIV3 MR1	CGC GGATCCATGATGCCCATATAACCAGA (BamHI)	4676–4695	
IDV	IDV-F	GATCGACTAGTCGCCACCATG acgtcaatgatatcattgaca(Spel I)	68–88	2135 bp
	IDV-R	GTTACTCGAGC ttcaattgcctctcccatcga (Xhol)	2150–2170	

### Optimization of the Multiplex Reaction System

**Figure 3:**
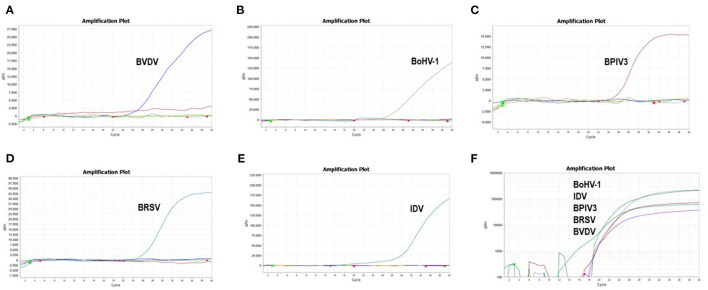
Specificity of the multiplex real-time PCR assay: The data were represented with amplification curves (X-axis: Cycle, Y-axis: ΔRn) **(A–E)** Amplification curves represent samples positive for BVDV, BoHV-1, BPIV3, BRSV and IDV detected by the multiplex real-time PCR assay. **(F)** Co-infection simulation experiments with nucleic acids of five pathogens extracted from cell culture The viruses can be detected by our multiplex real-time PCR.

**Figure 4:**
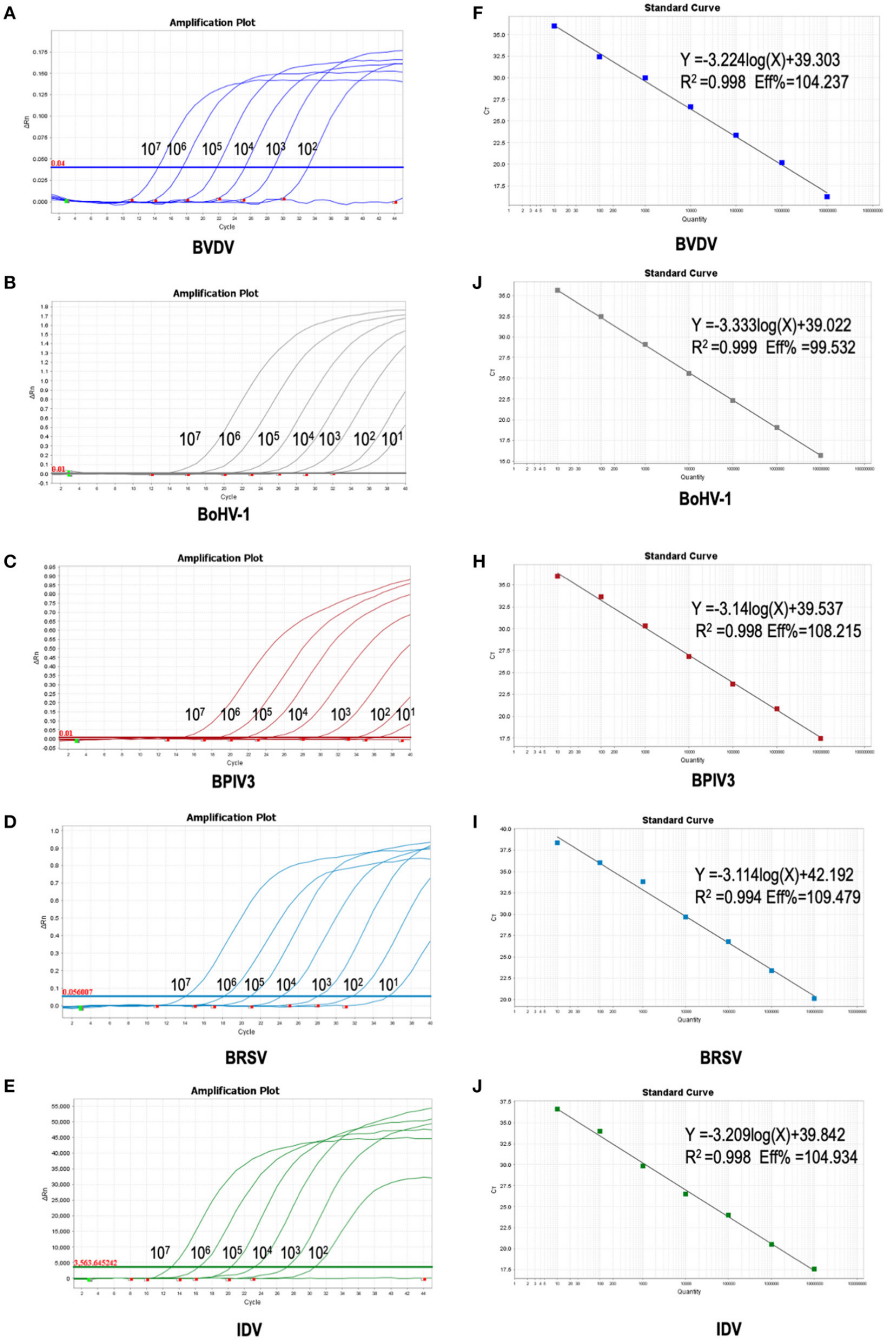
Sensitivity of the multiplex real-time PCR assay: **(A–E)** amplification curves (X-axis: Cycle, Y-axis: ΔRn) of plasmid standards of BVDV, BoHV-1,BPIV3,BRSV and IDV detected by multiplex real-time PCR. **(F–J)** standard curves of plasmid standards of BVDV, BoHV-1, BPIV3, BRSV and IDV. F: BVDV: Y = −3.224log(X)+39.303 R^2^ = 0.998 Eff% = 104.237; **(G)**: BoHV-1: Y = −3.333log(X)+39.022 R^2^ = 0.999 Eff% = 99.532; **(H)**: BPIV3: Y = −3.14log(X)+39.537 R^2^ = 0.998 Eff% = 108.215; **(I)**: BRSV: Y = −3.114log(X)+42.192 R^2^ = 0.994 Eff% = 109.479; **(J)**: IDV: Y = −3.209 log(X)+39.842 R^2^ = 0.998 Eff% = 104.934.

To improve the performance of the multiplex reaction system and reduce the interference among the fluorophores, the parameters including primer concentration, probe concentration, annealing temperatures, and cycling conditions were selected for optimization. The optimized multiple real-time PCR reactions were further evaluated by using the nucleic acids isolated from related pathogens including BVDV, BoHV-1, BPIV3, IDV, and BRSV. Amplification was carried out on Applied Biosystems QuantStudio 5 (Thermo Fisher Scientific). We found that a good amplification effect can be achieved when the total reaction system is 10 μL. Consistently, the optimized multiplex reaction system could efficiently recognize the specific pathogen with the corresponding primer/probe set (Figures 3A–F). By using the 10-fold serial diluted plasmid standards, we further validated the amplification efficiency and detection limits, and found that the sample with the concentrations as low as 1 × 10^1^ copies/μL are detectable. Unfortunately, this lowest number of nucleic acid copies per unit volume (1 × 10^1^ copies/μL) was not able to be detected in 95% of BVDV and IDV detection. Therefore, the lowest acceptance limit was defined as 1 × 10^2^ copies/μL for BVDV and IDV. The cutoff Ct value for BVDV positivity was defined at 36, which means the sample with a Ct value less than or equal to 36 (≤ 36) was considered as positive, but higher than 36(>36) are negative. The cutoff Ct value for BoHV-1 and BPIV3 positivity was defined at 36, and the cutoff Ct value for IDV positivity was defined at 37 (Figures 4A–E).

### Repeatability of the Multiplex Real-Time PCR Assay

To assess the repeatability of the multiplex real-time PCR assay, four dilutions of the plasmid standards ranging from 10^3^ to 10^6^ are used for intra-batch and inter-batch repetitions. The standard deviation and coefficient of variation were calculated based on the Ct values obtained from three replicates. As shown in Table 3, the coefficient of variation is <3%, indicating the method has good repeatability.

**Table 3:** Repeatability of the multiplex real-time PCR assay.

**Virus**	**Concentration**	**Intra-coefficient**		**Inter-coefficient**	
	**of template**	**of variation**		**of variation**	
	**(copies/μL)**				
		**X ±SD**	**CV(%)**	**X ±SD**	**CV(%)**
BVDV	10^6^	19.323 ± 0.134	0.70	19.319 ± 0.227	1.17
	10^5^	23.188 ± 0.016	0.07	23.140 ± 0.153	0.66
	10^4^	26.157 ± 0.044	0.17	26.411 ± 0.366	1.39
	10^3^	29.904 ± 0.123	0.41	29.598 ± 0.281	0.95
BOHV-1	10^6^	18.807 ± 0.034	0.18	18.874 ± 0.136	0.72
	10^5^	22.414 ± 0.038	0.17	22.428 ± 0.042	0.19
	10^4^	26.785 ± 0.089	0.33	26.727 ± 0.137	0.51
	10^3^	29.617 ± 0.179	0.60	29.428 ± 0.315	1.07
BRSV	10^6^	21.283 ± 0.038	1.70	21.759 ± 0.465	1.45
	10^5^	25.067 ± 0.032	0.12	25.177 ± 0.084	0.33
	10^4^	28.681 ± 0.023	0.80	28.852 ± 0.45	1.56
	10^3^	32.118 ± 0.125	0.39	33.081 ± 1.14	2.14
BPIV3	10^6^	20.815 ± 0.064	0.31	20.694 ± 0.362	1.75
	10^5^	24.435 ± 0.012	0.53	24.348 ± 0.559	2.29
	10^4^	27.510 ± 0.438	0.25	27.400 ± 0.285	1.04
	10^3^	30.747 ± 0.028	0.09	30.640 ± 0.288	0.94
IDV	10^6^	19.278 ± 0.042	0.21	19.425 ± 0.55	2.80
	10^5^	22.185 ± 0.349	1.57	22.650 ± 0.104	0.45
	10^4^	25.070 ± 0.074	0.30	25.608 ± 0.389	1.50
	10^3^	28.230 ± 0.099	0.35	28.664 ± 0.243	0.85

### Clinical Sample Detection

**Figure 5:**
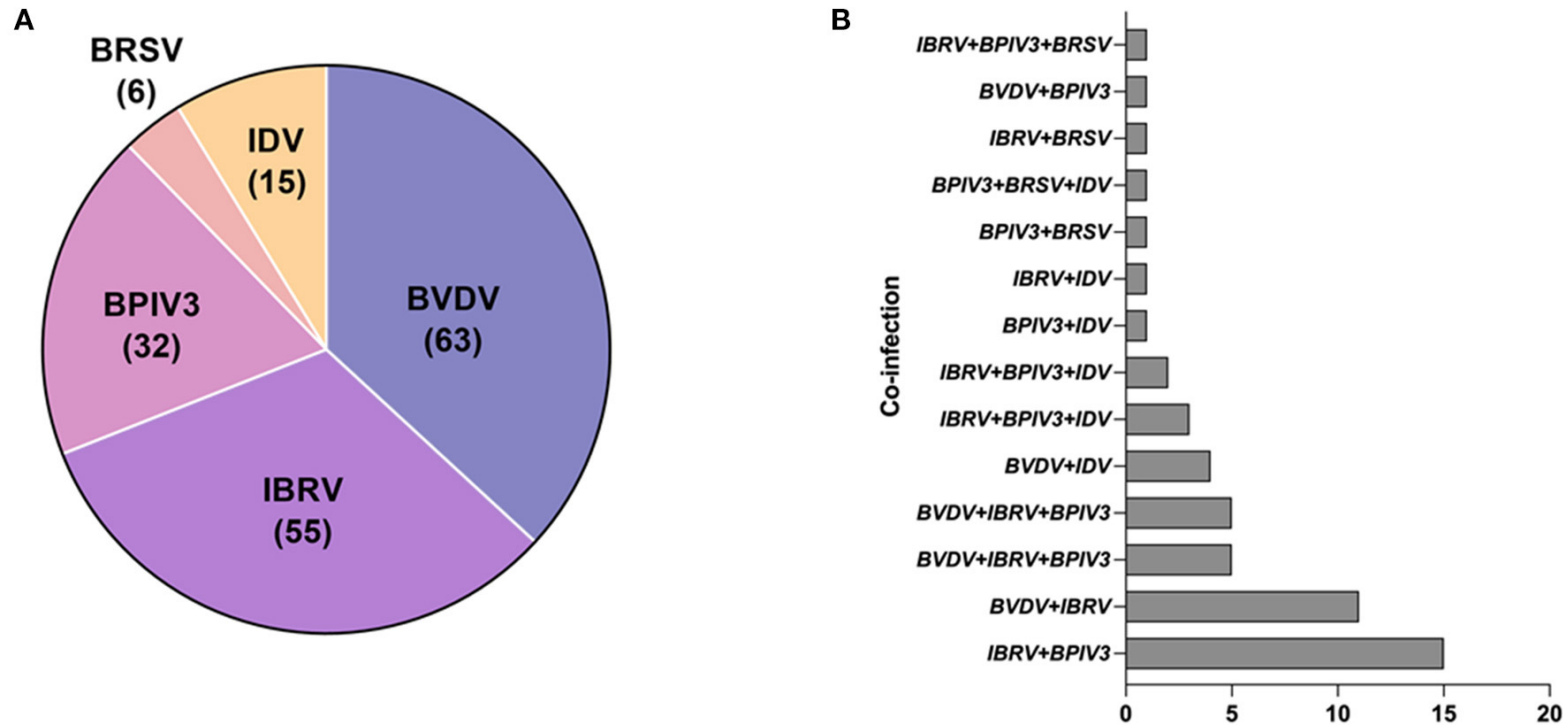
Clinical sample detection: **(A)** The distribution of clinical samples that are positive for BVDV, BoHV-1, BPIV3, BRSV or IDV. **(B)** The co-infection incidence among BVDV, IBRV, BPIV3, BRSV and IDV.

To further verify its clinical applicability for the differential diagnoses of BRDC associated viral pathogens, 213 clinical samples collected from the cattle with clinical signs of BRDC were tested. Among them, 63 (29.577 %) were detected as positive for BVDV, 55 (25.822 %) were detected as positive for BoHV-1, 32 (15.023 %) were detected as positive for BPIV3, 6 (2.817 %) were detected as positive for BRSV. Interestingly, 15 (7.042 %) IDV positive sample was also detected (Figure 5). Among all samples, 52 (24.413 %) clinical specimens were co-infected with two or more than two pathogens. Of note, 26(12.207 %) clinical samples were co-infected with BoHV-1and BPIV3, among which the co-infections of BPIV3 and BoHV-1 were the most serious (Table 4), indicating that BVDV and BoHV-1 is widespread in the dairy herds in China and exist serious co-infections.

**Table 4:** The number of clinical specimens with co-infection.

**Co-infection**	**Number**
BoHV-1, BPIV3, IDV	3
BVDV, BoHV-1, BPIV3	5
BVDV, BoHV-1, BPIV3	5
BoHV-1, BPIV3	15
BPIV3, IDV	1
BoHV-1, IDV	1
BVDV, BoHV-1	11
BPIV3, BRSV	1
BVDV, IDV	4
BPIV3, BRSV, IDV	1
BoHV-1, BRSV	1
BVDV, BPIV3	1
BoHV-1, BPIV3, BRSV	1

## Discussion

BRDC is an important disease that plagues the cattle industry and causes significant economic losses (1). Since BRDC is a muti-factorial disease, involving both viral and bacterial pathogens (26). The early diagnosis and detection of BRDC is of great economic and clinical significance (27). At present, the commonly used detection assays are classified into antigen detection and antibody detection (28). Conventional RT-PCR and PCR have been widely applied for pathogen detection. However, this method is only suitable for infection by a single pathogen, and time-consuming (29, 30). As bovine respiratory disease syndrome is mainly caused by co-infection of multiple pathogens, it is urgent to develop a rapid and convenient detection method to simultaneously the pathogens causing BRDC. So far, a variety of detection methods have been developed for the BRDC diagnosis (3, 21). For instance, Liu et al. developed a nanoparticle-assisted PCR assay for detection of BRSV, BoHV-1, BVDV, and BPIV3 with a detection limit of 1.43 × 10^2^ copies of recombinant plasmids per reaction (31). Leenadevi et al. developed a one-step multiplex real time RT-PCR for the detection of BRSV, BoHV-1 and BPIV3. The assay was rapid, highly repeatable, specific and had a sensitivity of 97% in detecting 10^2^ copies of BRSV, BoHV-1 and BPIV3 (22). Mari et al. developed a multiplex real-time RT-PCR assay for BVDV-1, BVDV-2 and HoBi-like pestivirus. The assay was found to be sensitive, specific and repeatable, ensuring detection of as few as 10^0^-10^1^ viral RNA copies (32). Although these methods have good specificity and sensitivity, the detected pathogens were only limited to BVDV, BoHV-1, or BPIV3. The accumulating studies have found that IDV and BRSV are also highly associated with bovine respiratory disease syndrome (19, 27). Therefore, the assays that are capable of detecting all these five pathogens are needed. In this study, primers and probes that could specifically recognize the dedicated pathogens were designed in the highly conserved regions. To improve the amplification efficiency of different primers and probes, the annealing temperature of the primers was set at 57°C, the annealing temperature of the probe was set at 67°C. Notably, in this developed assay, the primer/probe set of BVDV showed the lowest amplification. One possible explanation is that the fluorescence of Cy5 was the weakest and very susceptible to interference from other fluorophores due to its own physical properties (30). Despite the optimization such as primer/probe concentration and annealing temperature, the improvement effect was not significant. Among all these viruses, BoHV-1exhibited the highest amplification efficiency probably due to its double-stranded DNA structure which enables the amplification more efficient.

In the clinical samples tested, the highest prevalence was observed for BoHV-1 and BVDV, while the infection rate of BRSV is relatively lower. Our observations were consistent with the previous reports showing the prevalence of BoHV-1 and BVDV in China was ~40 and 50.10%, respectively (11, 33). As reported previously, the infection rate of BPIV3 and BRSV is relatively lower (34). However, a survey in Slovenia showed that the most prevalent virus was BRSV instead, followed by BCoV, BPIV3, and BVDV, while BoHV-1 was less frequently detected (35). The observed differences of BRSV and BoHV-1 prevalence indicated that the major causes of BRDC in different country/regions need to be determined by conducting routine surveillance. In addition, we found that the positive rate of IDV is more than 7.042%, suggesting that a certain prevalence of IDV occurs in the northeast part of China. The surveillance of IDV in cattle population in China needs to be performed.

Due to the sampling season and sampling method, the positive rate may be affected. As reported, the high incidence of respiratory diseases mainly occurs in spring (4). while the clinical samples used in this study were collected from the northeast part of China during 2019–2021. Notably, we found that the dual infections (BoHV-1 and BPIV3, BVDV and BoHV-1) were identified most often in the cattle with BRD symptoms. Due to the limited sample size, further investigation is required to verify the observation. In conclusion, this study established efficient multiple real-time PCR detection method which can detect the five major viral pathogens causing BVDV, BoHV-1, BRSV, BPIV3, and IDV, and provided support for the clinical surveillances.

## Data Availability Statement

The original contributions presented in the study are included in the article/supplementary material, further inquiries can be directed to the corresponding authors.

## Author Contributions

XY, YMZ, FX, and JZ designed the study, reviewed, and edited the manuscript. ZJ, WW, MJY, and JL collected the samples. ZJ performed the experiments. ZJ and XY wrote and edited the manuscript. All the authors read and approved the final manuscript.

## Funding

This work was supported by the Natural Science Foundation of Heilongjiang Province JQ2021C005 (XY). The funders had no role in study design, data collection and analysis, decision to publish, or preparation of the manuscript.

## Conflict of Interest

The authors declare that the research was conducted in the absence of any commercial or financial relationships that could be construed as a potential conflict of interest.

## Publisher's Note

All claims expressed in this article are solely those of the authors and do not necessarily represent those of their affiliated organizations, or those of the publisher, the editors and the reviewers. Any product that may be evaluated in this article, or claim that may be made by its manufacturer, is not guaranteed or endorsed by the publisher.
